# 2167. Mutational Signature Analysis to Predict Drug-resistant *Pseudomonas aeruginosa*

**DOI:** 10.1093/ofid/ofad500.1790

**Published:** 2023-11-27

**Authors:** Kalen M Hall, Leonard G Williams, Christine M Bojanowski, Zachary F Pursell, Lisa Morici

**Affiliations:** Tulane University School of Medicine, New Orleans, Louisiana; Tulane University School of Medicine, New Orleans, Louisiana; Tulane University School of Medicine, New Orleans, Louisiana; Tulane University School of Medicine, New Orleans, Louisiana; Tulane University School of Medicine, New Orleans, Louisiana

## Abstract

**Background:**

*Pseudomonas aeruginosa* (Pa) is one of the predominant bacteria that contributes to chronic respiratory infection, patient morbidity, and mortality in cystic fibrosis (CF) patients. Up to 60% of Pa clinical isolates are hypermutators, caused by deficiency in the DNA mismatch repair (MMR) system, and are associated with increased multidrug resistance and treatment failure. *In vitro* studies demonstrate a causative relationship between hypermutation and multidrug resistance, yet hypermutators are not currently tested for in the clinical setting as no diagnostic method is currently available. Mutational signatures have been computationally extracted and characterized in human tumors, but to our knowledge this analysis has never been performed in prokaryotes.

**Methods:**

Here, we evolved MMR-deficient and -proficient Pa *in vitro* under nine different antibiotic treatments. We sequenced these evolved lineages to test the hypothesis that mutational signature analysis can predict MMR-status and drug resistance. We identified mutation spectra from *de novo* mutations in the evolved lineages after ten passages and then compared these spectra to known human tumor MMR-deficient mutational signatures. We then sequenced 14 isolates from seven CF patients and performed mutational signature analyses to assess for the presence of MMR-deficient signatures.

**Results:**

We observed a strong signature in all evolved MMR-deficient Pa strains of C >T transitions in a NCC and NCG contexts with some 5’ preference to C and G, along with T >C transitions in CTN and GTN contexts. Although many antibiotics are known mutagens, we did not observe treatment-dependent changes in mutational spectra.Mutation spectra consistent with MMR-deficiency were found in four isolates, each of which were also functionally validated as hypermutant via rifampicin reversion assay.

**Conclusion:**

These studies suggest that mutational signature analysis may be a valuable tool to identify hypermutator strains of Pa, which could inform treatment regimens and improve management of chronic Pa infections.

**Disclosures:**

**Kalen M. Hall, B.S., B.A.**, Informuta: 1|Informuta: Ownership Interest|Informuta: Stocks/Bonds **Leonard G. Williams, B.S.**, Informuta: 1|Informuta: Ownership Interest|Informuta: Stocks/Bonds

